# Three Candidate Probiotic Strains Impact Gut Microbiota and Induce Anergy in Mice with Cow's Milk Allergy

**DOI:** 10.1128/AEM.01203-20

**Published:** 2020-10-15

**Authors:** Nathalie Esber, Aurélie Mauras, Johanne Delannoy, Chantal Labellie, Camille Mayeur, Marie-Aurore Caillaud, Toma Kashima, Landry Souchaud, Ioannis Nicolis, Nathalie Kapel, Anne-Judith Waligora-Dupriet

**Affiliations:** a UMR-S 1139, Université de Paris, INSERM, Paris, France; b UR 7537—BioSTM, Université de Paris, Paris, France; c Micalis Institute, AgroParisTech, INRAE, Université Paris-Saclay, Jouy-en-Josas, France; d Department of Coprology, APHP Sorbonne Université, Pitie-Salpetriere Hospital, Paris, France; The Pennsylvania State University

**Keywords:** probiotics, food allergy, cow’s milk allergy, murine model, gut microbiota, dendritic cells, anergy

## Abstract

Cow’s milk allergy is a worldwide public health issue, especially since there is no effective treatment, apart from milk and dairy product avoidance. The aim of this study was to assess the beneficial role of three probiotic strains previously selected for their prophylactic properties in a mouse model of β-lactoglobulin allergy. Administration of Lactobacillus rhamnosus LA305, L. salivarius LA307, or Bifidobacterium longum subsp. *infantis* LA308 for 3 weeks post–sensitization and challenge modified the composition of the gut microbiota, with an increase in the *Prevotella* NK3B31 group and a decrease in *Marvinbryantia*, belonging to the *Lachnospiraceae* family. Although no impact on markers of sensitization was detected, modifications of *foxp3*, *tgfβ*, and *il10* ileal gene expression, as well as plasma metabolomic alterations in the tryptophan pathway, were observed. Moreover, *ex vivo* studies showed that all probiotic strains induced significant decreases in cytokine production by β-lactoglobulin-stimulated splenocytes. Taken together, these results suggest that the three probiotic strains tested lead to alterations in immune responses, i.e., induction of a tolerogenic anergy and anti-inflammatory responses. This anergy could be linked to cecal microbiota modifications, although no impact on fecal short-chain fatty acid (SCFA) concentrations was detected. Anergy could also be linked to a direct impact of probiotic strains on dendritic cells, since costimulatory molecule expression was decreased following coincubation of these strains with bone marrow-derived dendritic cells (BMDCs). To conclude, all three candidate probiotic strains induced strain-specific gut microbiota and metabolic changes, which could potentially be beneficial for general health, as well as anergy, which could contribute to oral tolerance acquisition.

**IMPORTANCE** We showed previously that three probiotic strains, i.e., Lactobacillus rhamnosus LA305, L. salivarius LA307, and Bifidobacterium longum subsp. *infantis* LA308, exerted different preventive effects in a mouse model of cow’s milk allergy. In this study, we evaluated their potential benefits in a curative mouse model of cow’s milk allergy. When administered for 3 weeks after the sensitization process and a first allergic reaction, none of the strains modified the levels of sensitization and allergic markers. However, all three strains affected gut bacterium communities and modified immune and inflammatory responses, leading to a tolerogenic profile. Interestingly, all three strains exerted a direct effect on dendritic cells, which are known to play a major role in food sensitization through their potentially tolerogenic properties and anergic responses. Taken together, these data indicate a potentially beneficial role of the probiotic strains tested in this model of cow’s milk allergy with regard to tolerance acquisition.

## Abstract (precis)

We showed previously that three probiotic strains, i.e., Lactobacillus rhamnosus LA305, L. salivarius LA307, and Bifidobacterium longum subsp. *infantis* LA308, exerted different preventive effects in a mouse model of cow’s milk allergy. In this study, we evaluated their potential benefits in a curative mouse model of cow’s milk allergy. When administered for 3 weeks after the sensitization process and a first allergic reaction, none of the strains modified the levels of sensitization and allergic markers. However, all three strains affected gut bacterium communities and modified immune and inflammatory responses, leading to a tolerogenic profile. Interestingly, all three strains exerted a direct effect on dendritic cells, which are known to play a major role in food sensitization through their potentially tolerogenic properties and anergic responses. Taken together, these data indicate a potentially beneficial role of the probiotic strains tested in this model of cow’s milk allergy with regard to tolerance acquisition.

## INTRODUCTION

Cow’s milk allergy (CMA) is one of the most common allergic disorders in infants and young children, particularly in the industrialized world, where its prevalence reaches 2 to 7.5% in infants, without any marked variation over the past decade (1). CMA is manifested by a variety of symptoms and signs, including life-threatening anaphylaxis, and is commonly associated with stunted growth (2). Although it usually regresses by the age of 5 or 6 years, CMA is still a source of stress for families due to the need to follow a milk-free diet and can lead to subsequent nutritional deficiency if not treated appropriately. The World Allergy Organization Special Committee on Food Allergy has therefore identified CMA as an area in need of a rationale-based approach to a problem that is considered serious, with a worldwide public health impact, especially since there are no effective therapeutic measures apart from food avoidance and lengthy immunotherapy (3, 4).

Emerging evidence indicates that suboptimal establishment of the gut microbiota in early infancy represents a critical factor underlying the development of food allergy (FA) (5). Several birth cohort studies have shown an altered gut microbiota in infants with CMA versus healthy infants (6–8). Moreover, we and others have shown the protective role of a healthy microbiota and the deleterious role of a CMA-associated microbiota in humanized β-lactoglobulin (BLG)-sensitized mouse models (9, 10). Intestinal commensal bacteria and their sequential establishment are known to play a crucial role in the maturation of the intestinal immune system, modulation of the T-helper (Th) balance, acquisition of oral tolerance, and maintenance of gut wall epithelial integrity. These relationships between the intestinal microbiota, immune responses, and allergy have contributed to the concept of modulating the intestinal microbiota in order to prevent or manage allergic diseases, and they support the use of probiotics as antiallergic therapy. However, in the context of FA, and CMA in particular, most studies have evaluated the preventive impact of various probiotic strains, while only a few have investigated their curative properties, with conflicting results (11). We previously identified three probiotic strains (Lactobacillus rhamnosus LA305, L. salivarius strain LA307, and Bifidobacterium longum subsp. *infantis* LA308) with prophylactic properties in a murine model of food allergy (specifically allergy to BLG), in particular decreasing specific IgE responses and mast cell degranulation (12). Our objective in this study was to evaluate the impact of these three probiotic strains in a curative murine model of BLG allergy.

## RESULTS

### None of the three probiotic strains tested regulated markers of sensitization or mast cell degranulation.

**FIG 1:**
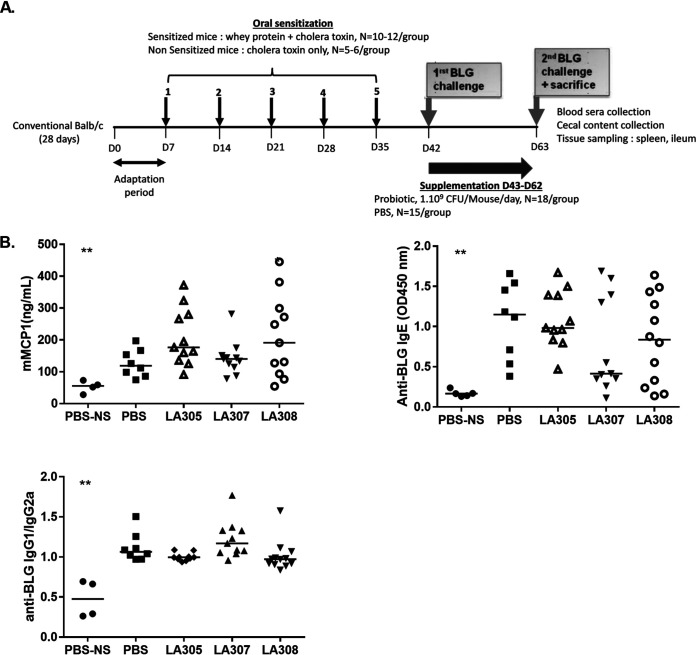
Impact of probiotic treatment on mast cell degranulation and BLG-specific antibody responses after an oral challenge with β-lactoglobulin (BLG). (A) Scheme of the experiment. D, day. (B) After the second BLG challenge, plasma mMCP-1 and anti-BLG-specific IgE levels, and the anti-BLG-specific IgG1/IgG2a ratio, were determined by ELISA. Each point represents a mouse, and the central horizontal line represents the median result for each group. Data were compared to those for WP-sensitized mice treated with PBS by using the Mann-Whitney U test, *, *P < *0.05; **, *P < *0.01.

The sequence of whey protein (WP) sensitization, challenge, and probiotic supplementation in our murine model of CMA is illustrated in Fig. 1A. Levels of BLG-specific IgE, expressing sensitization to WP, increased significantly during the five sensitization steps over those for the nonsensitized (NS) control group receiving cholera toxin (CT) alone (*P* < 0.01), reaching a median value on day 42 (D42) that corresponded to completion of the sensitization procedure (median optical density at 450 nm [OD_450_], 0.85 [range, 0.21 to 1.47]), similar to that observed at D63 after the second BLG challenge (median OD_450_, 1.15 [range, 0.38 to 1.66]). At D63, the end of the 20-day supplementation period, NS mice that received only CT showed low levels of mouse mast cell protease-1 (mMCP-1) and BLG-specific IgE, IgG1, and IgG2a in plasma, irrespective of whether they had been treated with probiotics. All WP-sensitized groups, whether treated with probiotics or not, showed significantly higher levels of mMCP1 and BLG-specific IgE, and higher IgG1/IgG2a responses, than those of the NS groups (*P < *0.01). With regard to WP-sensitized mice, no statistically significant differences in levels of mMCP-1 and BLG-specific IgE, or in the IgG1/IgG2a ratio, were observed between any of the three probiotic-treated groups and the phosphate-buffered saline (PBS)-treated control group (Fig. 1B).

### A 3-week supplementation with each of the three probiotic strains impacted gut microbiota in allergic mice without modifying short-chain fatty acid (SCFA) production.

**FIG 2:**
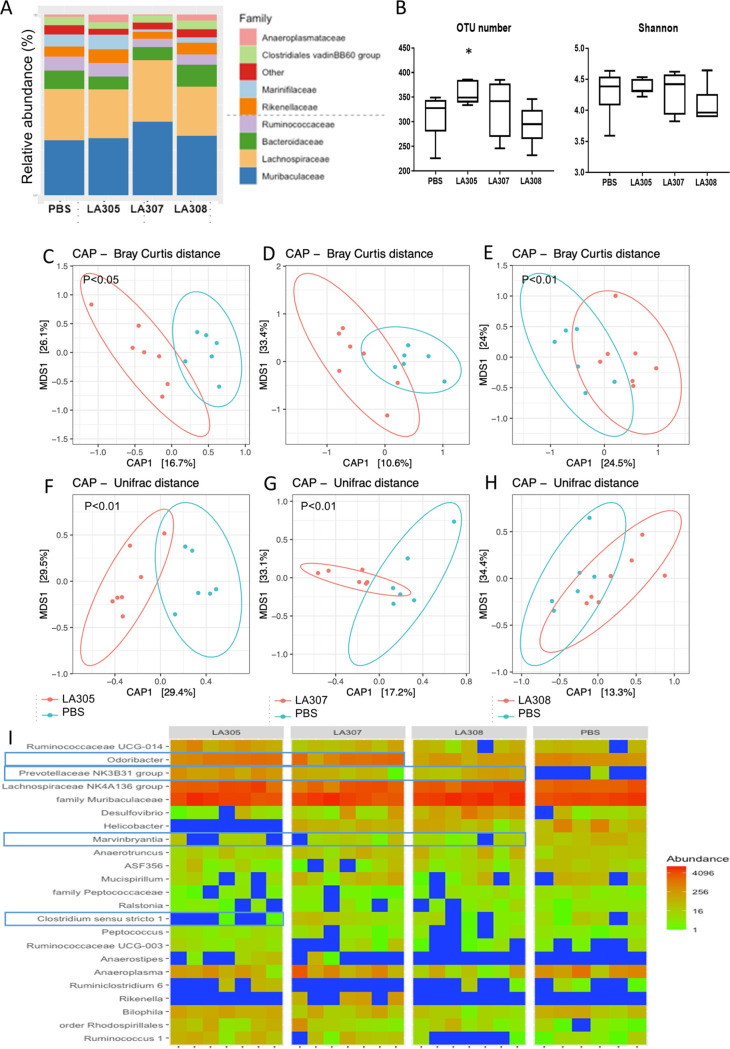
Microbiota analysis in sensitized mice. (A) Relative abundances of bacterial families in cecal samples (*n*, 6 to 7/group). (B) α-Diversity between the groups. The *y* axes show the numbers of species observed (left) and the Shannon diversity index (right). The box plots show the median (central horizontal line), the 25th percentile (lower box border), and the 75th percentile (upper box border). The lower and upper horizontal lines show the 10th and 90th percentiles, respectively. *, *P < *0.05. (C to H) β-Diversity observed with Bray-Curtis distance (C to E) and UniFrac distance (F to H). Scatterplots were generated using constrained analysis of principal coordinates (CAP). Mice that received PBS only are represented in blue. Probiotic groups are represented in red for LA305 (C and F), LA307 (D and G), and LA308 (E and H). β-Diversity was compared to that of WP-sensitized mice treated with PBS by use of PERMANOVA. (I) CAP-ordinated heat maps of cecal bacteria in the four groups of mice at the genus level. The genera indicated are derived from SIMPER and LefSe analyses and discriminated one or several of the groups receiving probiotics from the group receiving PBS.

Microbiota analyses were performed on cecal contents (CC) at D63 using 16S rRNA gene sequencing. A total of 624 operational taxonomic units (OTUs) were identified. Among these, we selected for further analysis 454 OTUs, comprising the 99% most abundant OTUs, belonging to 7 phyla, 22 families, and 64 genera. Analyses were performed at the genus level. The three probiotic strains modified the microbiota in different ways (Fig. 2A). Only treatment with L. rhamnosus LA305 after the first BLG challenge significantly increased microbiota richness (*P* < 0.05), increasing the number of OTUs over that for the PBS-treated group (Fig. 2B). However, these modifications did not affect α-diversity expressed as the Shannon index (Fig. 2B). Supplementation with LA305 led to a modification in dominant, phylogenetically unrelated genera, as well as in subdominant genera, as evidenced by the fact that β-diversity differed significantly between the PBS- and LA305-treated groups whether calculated according to Bray-Curtis (Fig. 2C), UniFrac (Fig. 2F), or Jaccard (data not shown) distances. Treatment with L. salivarius LA307 did not affect α-diversity, but the composition of phylogenetically unrelated subdominant genera was modified, as evidenced by the fact that β-diversity differed significantly between the PBS- and LA307-treated groups when calculated on the basis of UniFrac (Fig. 2G) and Jaccard (data not shown) distances but not when calculated according to Bray-Curtis distance (Fig. 2D). In contrast, B. longum subsp. *infantis* LA308 altered only dominant, phylogenetically related genera, as evidenced by the fact that β-diversity differed significantly from the PBS group only when calculated according to Bray-Curtis distance (Fig. 2E) and not when calculated according to UniFrac distance (Fig. 2H).

The numbers of genera that significantly differentiated the microbiota of probiotic-treated mice from that of PBS-treated mice were 17, 4, and 8 for LA305, LA307, and LA308, respectively (Fig. 2I; see also Fig. S1 and Table S1 in the supplemental material). Two genera were similarly affected by all three probiotic strains: the *Prevotellaceae* NK3B31 group was increased, and the *Marvinbryantia* genus, belonging to the *Lachnospiraceae* family, was decreased. The microbiotas of LA305- and LA307-treated mice were enriched in *Odoribacter* spp., whereas levels of this genus were lower in the microbiota of LA308-treated mice than in the PBS group. Other modifications unique to a particular probiotic strain were observed, confirming the strain-specific impact of probiotics on the microbiota. For instance, apart from that of *Marvinbryantia*, LA305 modified the abundances of several genera belonging to the *Firmicutes*, increasing those of some *Peptococcaceae* and *Ruminococcaceae*, and decreasing that of *Clostridium sensu stricto*. It also decreased the abundances of *Deferribacteres* (*Mucispirillum* spp.), *Epsilonbacteraeota* (*Helicobacter* spp.), and *Proteobacteria* (*Ralstonia* spp. and *Rhodospirillales*). In contrast, LA308 increased the abundance of the *Lachnospiraceae* NK4A136 group, altered the composition of *Proteobacteria* (decreasing *Bilophila* spp. and increasing *Desulfovibrio* spp.), and decreased the abundance of *Anaeroplasma*. It should be noted that our analyses did not show any modification in species of *Lactobacillus*, a major genus within the mouse intestinal microbiota, or in *Bifidobacterium* spp., which are at low levels, regardless of the probiotic strain administered.

Despite these modifications in the gut microbiota, fermentative activities and short-chain fatty acid production after probiotic treatment were not modified from that with PBS treatment, irrespective of the strain tested (Fig. S2).

### All three probiotic strains decreased *ex vivo* secretion of T-helper cell cytokines in response to allergen challenge.

**FIG 3:**
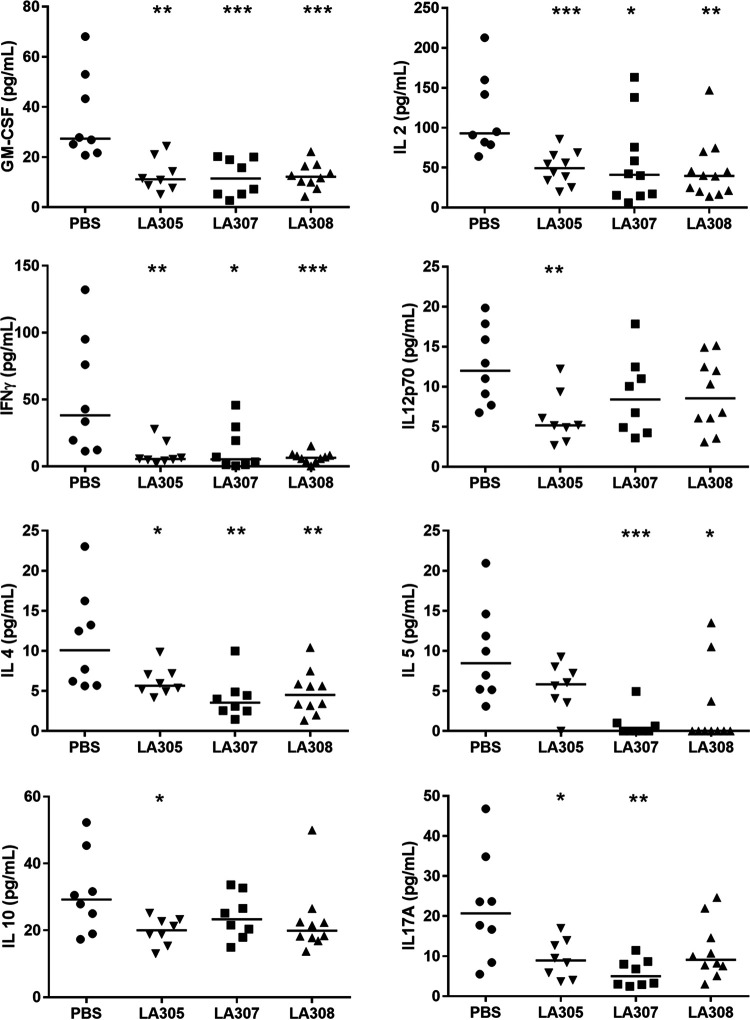
Impact of probiotic treatment on *ex vivo* cytokine secretion by BLG-stimulated splenocytes. Splenocytes isolated at D63 from WP-sensitized mice were rechallenged *ex vivo* for 2 days in the presence of BLG (2.5 mg/ml). Each point represents a mouse, and the central horizontal line represents the median of each group. Cytokine concentrations in culture supernatants of splenocytes from probiotic-treated WP-sensitized mice versus PBS controls were compared using the Mann-Whitney U test (*, *P < *0.05; **, *P < *0.01; ***, *P < *0.001).

The T-helper response to antigen challenge was evaluated by measuring the quantities of cytokines secreted into culture supernatants by splenocytes isolated from WP-sensitized mice supplemented with the three probiotics tested or PBS (controls) and cultured *ex vivo* in the presence or absence of BLG. Splenocytes cultured in the absence of BLG secreted very small amounts of cytokines (data not shown) in contrast to splenocytes from PBS-treated mice exposed to BLG challenge. Cytokine secretions by splenocytes isolated from mice that had received probiotic supplementation were lower than those by splenocytes isolated from control mice that had received only PBS, suggesting a possible anergic effect of the probiotics. In particular, splenocytes isolated from mice treated with each of the three probiotics secreted significantly lower quantities of granulocyte-macrophage colony-stimulating factor (GM-CSF), gamma interferon (IFN-γ), interleukin 2 (IL-2), and IL-4 than splenocytes from PBS controls (Fig. 3). Decreases in the secretion of other cytokines were strain specific. Significant decreases in the secretion of IL12p70 (*P* < 0.01) and IL-10 (*P* < 0.05) were observed in the culture supernatants of splenocytes isolated from LA305-treated mice, whereas no effect on IL-5 levels was observed. In contrast, splenocytes isolated from LA307-treated mice secreted significantly less IL-5 (*P* < 0.001) and IL17A (*P* < 0.01), whereas no effect on IL-10 was seen. Splenocytes isolated from LA308-treated mice showed the same profile of decreases in cytokine secretion as those from LA307-treated mice except for a lack of effect on IL17A secretion (Fig. 3).

### The three probiotic strains tested had different effects on T-helper cell transcription pathways in the ileum.

**FIG 4:**
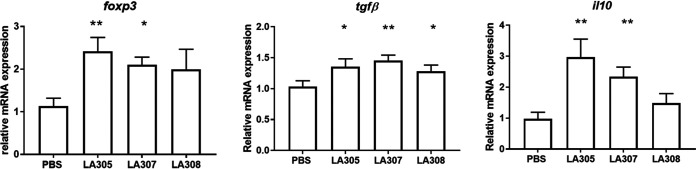
Impact of probiotic treatment on the ileal local immune response profile. The expression of ileal gene mRNA (*foxp3*, *tgf*β, and *il10*) in sensitized mice (*n*, 12 for each probiotic group) was quantified by quantitative real-time PCR and normalized to expression for the sensitized PBS control group (*n* = 10). Data are expressed as means ± SEM. Data were compared to those for PBS-treated WP-sensitized mice using the Mann-Whitney U test (*, *P < *0.05; **, *P < *0.01).

The effect of probiotic treatment on T-helper cell pathways in the terminal ileum was evaluated through analyses of gene expression of cytokines and transcription factors related to the T-helper cell subsets Th1, Th2, and Th17, as well as regulatory T cells (Treg cells). None of the probiotics tested had a significant impact on T-helper cell gene expression in WP-sensitized mice (data not shown). However, all three probiotic strains affected the expression of genes linked to Treg cells and anergy (Fig. 4). Treatment with LA305 or LA307 significantly increased *foxp3* (*P*, <0.01 and <0.05, respectively), *tgfβ* (*P*, <0.05 and <0.01, respectively), and *il10* (*P*, <0.01 and <0.01, respectively) mRNA expression, while LA308 induced an increase only in *tgfβ* gene expression (*P* < 0.05).

### All three probiotic strains modified plasma kynurenic metabolism.

**FIG 5:**
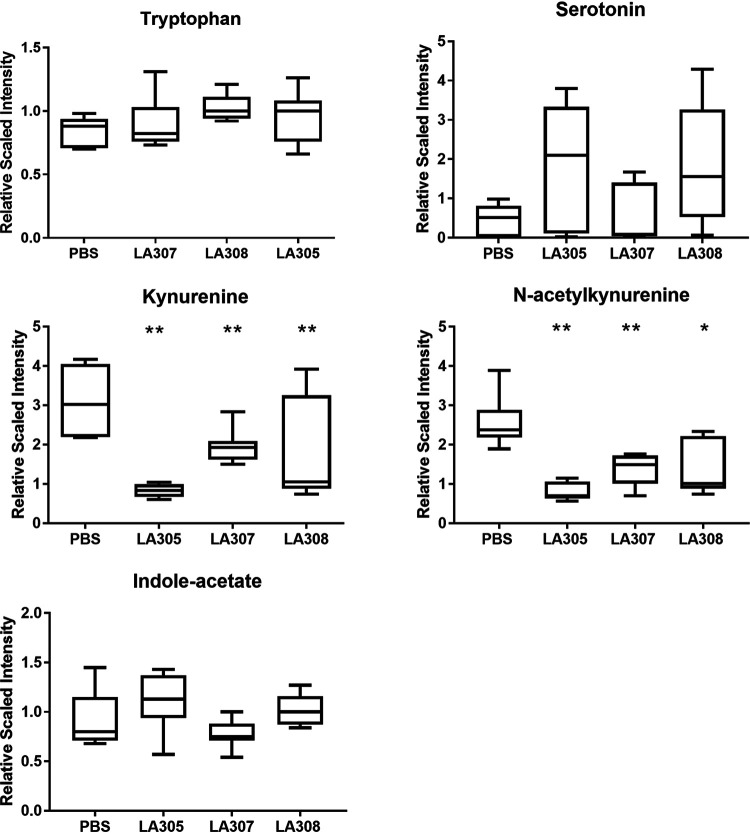
Impact of probiotic treatment on the tryptophan pathway. The metabolomes in plasma samples from WP-sensitized PBS-treated (*n* = 5) and probiotic-treated (*n*, 5 for each probiotic group) mice were analyzed by Metabolon using UPLC–MS/MS. To allow comparison of all biochemicals on a similar scale, the scaled imputed data method was used (i.e., the median value was set to 1, and all other values were scaled accordingly). The box plots show the median (central horizontal line), the 25th percentile (lower box border), and the 75th percentile (upper box border). The lower and upper horizontal lines show the 10th and 90th percentiles, respectively. Data were compared to those for PBS-treated WP-sensitized mice using the Mann-Whitney U test (*, *P < *0.05; **, *P < *0.01).

The effect of probiotic treatment on metabolic pathways was evaluated through metabolomic analyses of plasma samples. Seven hundred named biochemicals were detected. LA305, LA307, and LA308 significantly modified the abundances of 47, 30, and 19 of these compounds, respectively, from those with PBS treatment. The quantities of certain microbial metabolites usually found in plasma under normal conditions were modified by probiotic treatment; e.g., *p*-cresol sulfate was decreased 3.8-fold by LA305, and phenol sulfate showed a trend to decrease (1.7-fold) with LA308. Changes in the abundances of certain modified lysines and RNA-related biochemicals were observed with LA308, and the quantities of certain compounds implicated in the citric acid cycle pathway were modified by LA305 and LA308 supplementation. However, the physiological consequences of these changes, such as histone deacetylation or demethylation, were difficult to determine at this level. Analysis of the tryptophan pathway, known to be associated with tolerance acquisition, showed that all three probiotic strains induced significant decreases in kynurenine and *N*-acetylkynurenine levels from those with PBS treatment, while none of the probiotics affected the levels of tryptophan, serotonin, or indole-acetate in plasma (Fig. 5).

### All three probiotic strains impacted BDMC maturity.

**FIG 6:**
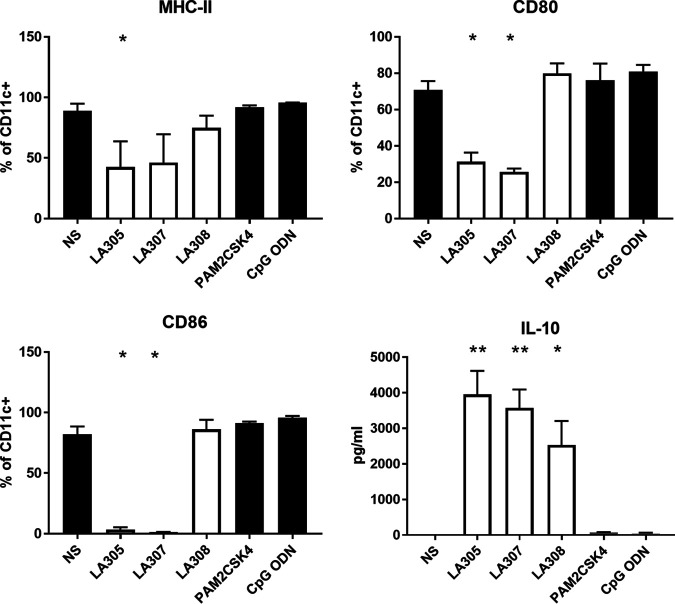
Impact of the three probiotic strains on BMDC maturation. Probiotic strains were coincubated with BMDCs (results from three experiments performed, each with three independent bacterial cultures). BMDCs without stimulation were used as an experimental control, and PAM2CSK4 (a TLR-2 ligand) and CpG-ODN (a TLR-9 ligand) were used as controls. The maturation of BMDCs was evaluated by FACS analysis: MHC-II, CD80, CD86. CD11c was used as a BMDC surface marker. The supernatant was collected, and interleukin-10 was measured by ELISA. Results are expressed as means ± SEM. Data were compared to those for nonstimulated BMDCs by using the Mann-Whitney U test (*, *P < *0.05; **, *P < *0.01).

Bone marrow-derived dendritic cells (BMDCs) isolated from BALB/cByJ mice were used to evaluate the immunomodulatory properties of the three probiotic strains, since these cells are a relevant model of DCs, as shown previously through their *in vitro* and *in vivo* interactions with T cells (13, 14). Stimulated BMDCs express costimulatory molecules on their surfaces, allowing them to present the processed antigens associated with major histocompatibility complex class II (MHC-II) molecules. In all our experiments, we targeted CD11c^+^ cells, which represented 80% ± 2.8% of total cells whatever the incubation conditions. Under basal conditions, approximately 80% of CD11c^+^ cells expressed CD86 and MHC-II, and 70% expressed the costimulatory molecule CD80 (Fig. 6). Coincubation with PAM2CSK4, which is a Toll-like receptor 2 (TLR-2) agonist, or with CpG oligodeoxynucleotide (ODN), which is a TLR-9 agonist, did not alter this expression profile. Following an 18-h coincubation period, LA305 induced significant decreases in MHC-II (2-fold), CD80 (2.2-fold), and CD86 (24-fold) expression on BMDCs. LA307 induced the same expression profile with 1.9-fold, 2.7-fold, and 59-fold decreases in MHC-II, CD80, and CD86 expression, respectively. In contrast, LA308 did not significantly modify the expression of costimulatory molecules on BMDCs. However, all three strains significantly increased IL-10 secretion by BMDCs (*P*, <0.01 for LA305 and LA307 and <0.05 for LA308). Such increased secretion was not observed on analysis of the culture supernatants of unstimulated BMDCs or BMDCs coincubated with TLR-2 and TLR-9 agonists.

## DISCUSSION

In this study, we showed that the postchallenge administration of three candidate probiotic strains, two belonging to the *Lactobacillus* genus and one to the *Bifidobacterium* genus, for 3 weeks in a murine model of CMA induced gut microbiota and immune response alterations, i.e., tolerogenic anergy and low inflammatory status, although no impact on serum markers of sensitization was observed. The three probiotic strains, L. rhamnosus LA305, L. salivarius LA307, and B. longum subsp. *infantis* LA308, had been selected in a previous study out of a panel of 31 strains for their prophylactic properties (12). In the present study, we evaluated their therapeutic potential in a murine model of CMA. Sensitization of mice with WP was previously shown to induce high levels of specific IgE and IgG1 antibodies, and a substantial release of mMCP-1 was observed following allergen challenge (12). In the study reported here, none of the three probiotic strains modified this natural evolution when administered daily after the first challenge. This result could have been due to the short duration of administration compared to IgG1- and IgE-producing cell half-lives (15, 16). However, a similar study using ovalbumin instead of WP for sensitization and administration of a synbiotic mixture of nondigestible oligosaccharides and Bifidobacterium breve M-16V reported beneficial results on allergy markers (17). In the model described here, the initial BLG challenge performed at D42, i.e., before the administration of probiotics, might have modified the allergic response at D63. Despite the absence of beneficial effects on the serologic markers of sensitization and allergy, the 20-day supplementation performed after the first challenge led to a marked decrease in the secretion of all cytokines when splenocytes were subjected to allergen stimulation *ex vivo*. Such a reduction of allergen-specific Th2 cytokine production has been already described after oral immunotherapy and is attributed mainly to T-cell anergy, i.e., T-cell unresponsiveness to the antigen (18). The three probiotic strains were thus able to induce anergy, a mechanism known to contribute to oral tolerance (19). Anergy is a mechanism supported by recent human studies for oral tolerance. Its induction could be related to the stimulation of Treg cells. Indeed, supplementation with either LA305 or LA307 induced an increase in ileal expression of *foxp3*, *tgfβ*, and *il10*. However, the mechanism by which the three strains could induce Treg-cell development is not clear. Treg-cell induction could be related to the production of immunoregulatory metabolites, particularly acetate, propionate, and butyrate, by the intestinal microbiota (20), since SCFAs have previously been proposed to suppress food allergy by eliciting protective mucosal Treg-cell responses and enhancing intestinal barrier integrity (21). However, we did not detect any significant variation in SCFA concentrations after probiotic treatment whatever the strain used, although all three probiotic strains impacted gut microbiota, including an enrichment of *Odoribacter* spp. following LA305 and LA307 treatment, Odoribacter splanchnicus being a known producer of acetate, propionate, and butyrate (22). Similar results have been reported by Abdel-Gadir et al., who found no correlation between the production of SCFAs by bacterial consortia or SCFA oral administration and their efficacy in treating food allergy in mice (23). Another potential link between the composition of the gut microbiota and Treg induction relies on the ability of the microbiota to regulate the tryptophan pathway, which is associated with a protolerogenic Treg-cell response through the production of a variety of bioactive compounds, such as kynurenine (24). We did not detect any increase in tryptophan metabolites such as kynurenine or acetyl-kynurenine, but a decrease. Georgin-Lavialle et al. reported a significant decrease in the serum tryptophan level associated with an increase in the kynurenine level and indoleamine 2,3-dioxygenase (IDO) activity in patients with mastocytosis associated with digestive symptoms compared to healthy controls and patients without such symptoms (25). In our model, the sensitization process leads transiently to digestive symptoms (9); the decrease of the kynurenine pathway observed following probiotic administration could therefore be a marker of improved digestive health related to less inflammation in the probiotic-treated groups.

Since we did not identify any argument suggesting the implication of classical pathways with active suppressive Treg-cell induction by the microbiota, we focused our research on another mechanism of T-cell anergy. Indeed, reduction of allergen-specific Th2 cytokine production observed after oral immunotherapy for food allergy has been attributed mainly to T-cell anergy, while the generation of Treg cells in this process is not clear (18, 26). Interestingly, the *Prevotella* NK3B31 group was increased in the three probiotic-treated groups of mice. A decrease in *Prevotella* spp. has been reported in allergic infants and children (27), and an increase in this bacterial group was associated with the protective effect of prebiotic treatment in a CMA mouse model (28). Moreover, Prevotella intermedia has been demonstrated to have immunosuppressive effects on *in vitro* human lymphocyte activation, inhibiting the release of cytokines and expression of the interleukin 2 receptor and CD69 on T cells (29), a mechanism of action in accordance with our observations. Similarly, a significant increase in *Lachnospiraceae* has been reported in children with CMA (8), and *Marvinbryantia*, a genus belonging to the *Lachnospiraceae* family, was decreased by all three probiotic strains tested in our study. Although no specific mechanism could be determined based on these data, our principal aim was to assess the direct role of these strains.

Anergy can be induced by dendritic cells. The impact of these key players in the acquisition of tolerance depends on their cytokine production pattern and expression of costimulatory molecules (30). Following *in vitro* coincubation with BMDCs, the two lactobacillus strains decreased MHC-II, CD80, and CD86 costimulatory molecule expression at the surfaces of dendritic cells. These cells are in a semimature state and can exert tolerogenic effects via the induction of anergy (31). Indeed, anergy is induced following recognition of the antigen by T cells in the absence of costimulatory signals, that is, the binding of CD28 on their surfaces to its ligand, CD80/CD86 on dendritic cells (32). Moreover, in our study, coincubation with LA305 and LA307 induced a pronounced increase in production of the regulatory cytokine IL-10 by dendritic cells. While it had no effect on costimulatory molecules on BMDCs, LA308 also induced a similar increase in IL-10 production.

In our previous study, using a prophylactic mouse model, the three probiotic strains had distinct immune profiles. LA307 was rather immunosuppressive, with no impact on IFN-γ and IL-10 production, and could stimulate Treg cells. LA308 increased the production of Th1 cytokines, which inhibit the proliferation of Th2 cells, whereas LA305 had pro-Th1 and regulatory effects (12).

In this study, we show that the three probiotic strains administered postsensitization in our curative model of CMA led to modification of the gut microbiota (LA305 having a major impact compared to the other two strains) as well as to the development of tolerogenic anergy. Impact on gut microbiota is a proof of concept, since the mouse gut microbiota could not be compared to the human gut microbiota. However, we speculate that several modifications of the microbiota induced by LA305 could reinforce this effect. *Ralstonia* spp., known for their proinflammatory effects, were depleted, as previously observed by Fu et al. following *Lactobacillus* treatment in another CMA mouse model (33). Similarly, *Clostridium sensu stricto*, a genus that has been associated with the microbiota of allergic children harboring an IgE-dependent food allergy or atopic dermatitis (34, 35), was also decreased. In contrast, LA308 belongs to the *Bifidobacterium* group, a bacterial genus that is not as well adapted as lactobactobacilli to the mouse gut ecosystem. This could explain why this strain had an impact on the microbiota and metabolome different from those of the two *Lactobacillus* strains.

**FIG 7:**
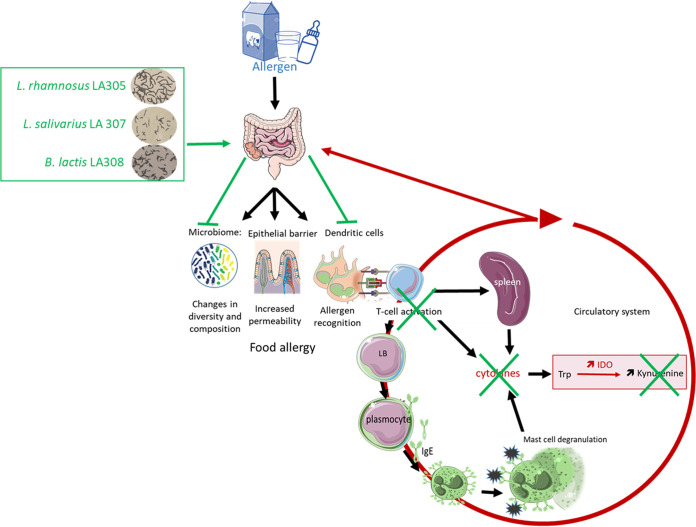
Overview of the proof of concept. Administration of one of the probiotic strains modulates the composition and diversity of the intestinal microbiota and downregulates the expression of costimulatory molecules on the surfaces of dendritic cells. These cells are in a state of semimaturity and do not activate helper T cells, resulting in anergy, with a decrease in the production and secretion of the cytokines involved in the allergic reaction. In particular, the decrease in proinflammatory cytokines, which are known to activate IDO, leads to a decrease in kynurenine, a marker of inflammation, in the general circulation. The three probiotic strains could then play a role in the acquisition of oral tolerance.

In conclusion, oral administration of the three candidate probiotic strains to mice with induced allergy to BLG led to microbial and metabolic changes that could potentially be beneficial for general health, as well as to anergy, which could play a role in oral tolerance acquisition (Fig. 7). Immunotherapy is recommended for children with persistent CMA over the age of 4 to 5 years but has been associated with significant risks (36). Therefore, these probiotic strains could be useful as adjuvants in desensitization processes, as reported for a strain of L. rhamnosus combined with oral immunotherapy of peanut allergy (37). Clinical studies would have to be performed to confirm these experimental results and to determine which is the most effective of the three probiotic strains tested.

## MATERIALS AND METHODS

### Animals and housing conditions.

Three-week-old conventional female BALB/cByJ mice were purchased from Charles River Laboratories (CRL; L’Arbresle, France). Mice were housed under conventional conditions in adapted and enriched cages maintained under a 12-h light-dark cycle, in temperature- and humidity-controlled chambers. They had free access to water and mouse pellets lacking cow’s milk proteins (A03; SAFE, Augy, France). All animal experiments began after 1 week of adaptation. All procedures were carried out in accordance with European guidelines for the care and use of laboratory animals. The protocol was approved by the Regional Council of Ethics for animal experimentation (Ile de France-Paris Descartes, CEEA34.AJWD.062.12). Experiments were performed in the animal care facilities of the CRP2-UMS 3612 CNRS-US25 INSERM-IRD unit in the Faculty of Pharmacy of Paris Descartes University, Paris, France.

### Mouse model of β-lactoglobulin food allergy: sensitization and probiotic administration.

Our model was adapted from the work of Li et al. (38). After an adaptation period, female BALB/cByJ mice were either sensitized once a week for 5 weeks by intragastric administration of WP (15 mg/mouse; Lacprodan 80; Arla, Lyon, France) and CT (10 μg/mouse; List Biological, Campbell, CA) suspended in 100 μl of PBS (sensitized group) or treated with 10 μg CT alone in PBS (nonsensitized group) (Fig. 1). On D42, an initial challenge was performed by oral administration of 60 mg of BLG (Sigma-Aldrich, France). On the following day, to avoid a cage effect associated with the sensitization process, the mice were randomly assigned to new cages and either to one of the three probiotic groups or to the PBS control group. Each of these groups included two subgroups: sensitized mice (*n*, 12 in each probiotic group and 10 in the PBS group) and nonsensitized mice (*n*, 6 in each probiotic group and 5 in the PBS group). In the probiotic groups, mice received daily, for 20 days (from D43 to D62), a single lyophilized bacterial strain (10^9^ CFU), either Lactobacillus rhamnosus LA305, L. salivarius LA307, or Bifidobacterium longum subsp. *infantis* LA308 (Genibio, Lorp-Sentaraille, France), suspended in 200 μl of PBS. The dose we chose to use is the one we used to demonstrate the preventive effect of these three strains on BLG allergy (12). Gavage was performed by two researchers who rotated between cages every 2 days. Mice in the control group received 100 μl of sterile PBS. On D63, all mice received a second oral challenge comprising administration of 60 mg of BLG (Fig. 1A).

### Measurements of mast cell protease-1 and BLG-specific IgE, IgG1, and IgG2 antibodies in plasma.

Blood from each mouse was collected in K_3_-EDTA tubes 45 min after the second BLG challenge. The blood samples were immediately centrifuged (at 3,000 rpm for 10 min at 4°C), and plasma samples were stored at −80°C until use. Concentrations of mouse mast cell protease-1 (mMCP-1) in plasma were measured by an enzyme-linked immunosorbent assay (ELISA) (Mouse MCPT-1 [mMCP-1] ELISA kit; Invitrogen, Thermo Scientific, Illkirch, France) according to the manufacturer's instructions. BLG-specific IgE, IgG1, and IgG2a levels were measured as described previously (39). Briefly, BLG-specific IgE levels were measured using a rat anti-mouse IgE antibody (Pharmingen, BD Biosciences, Le Pont-de-Claix, France) as the capture antibody and biotinylated BLG (Pierce, Rockford, IL) and streptavidin-horseradish peroxidase (HRP) (CliniSciences, Nanterre, France) as the detecting system. BLG-specific IgG1 and IgG2a were measured using BLG as the capture antigen and goat anti-mouse IgG1-HRP and IgG2a-HRP (CliniSciences, Nanterre, France) as the detecting antibodies. All analyses were performed in duplicate, and the results were expressed in terms of OD read at 450 nm. The IgG1/IgG2a ratio was calculated as a reflection of Th2/Th1 reactivity.

### Measurement of cytokine production by BLG-stimulated splenocytes.

Spleens were collected on the day of mouse sacrifice and were placed in sterile RPMI 1640 medium. Then splenocytes were isolated and cultured *ex vivo* as described previously (40). Briefly, spleens were gently crushed and filtered through a 70-μm nylon filter (Falcon; Dutcher, Brumath, France). After elimination of red blood cells, splenocytes were resuspended in RPMI 1640 complete tissue culture medium (Gibco, Thermo Scientific, Illkirch, France) containing 1% penicillin-streptomycin, 2 mM l-glutamine, and 10% fetal calf serum. Splenocytes were cultured in 24-well plates at a density of 2 × 10^6^ per well, with or without 2.5 mg/ml BLG at 37°C under an atmosphere of 5% CO_2_ and 95% air. Culture supernatants were collected after 48 h and stored at –80°C until analysis.

Levels of IL-17A, IL-2, GM-CSF, IL-4, IFN-γ, IL-10, IL-5, and IL-12p70 in splenocyte culture supernatants were quantified using Bio-Plex immunoassay kits (Bio-Plex Pro cytokine panel and Luminex Bio-Plex 200 system; Bio-Rad, Life Science Group, Hercules, CA) according to the manufacturer’s instructions.

### Quantification of ileal gene expression by quantitative real-time PCR.

On the day of mouse sacrifice, the ileum of each mouse was removed and stored in RNAlater at –80°C until RNA extraction. Total RNA was isolated from a 1-cm terminal ileum segment devoid of Peyer’s patches by using an RNeasy Plus universal minikit (Qiagen, Courtaboeuf, France) according to the manufacturer's instructions. RNA was then treated with DNase I (Invitrogen, Thermo Scientific, Illkirch, France), and first-strand cDNA was synthesized from 500 ng of total RNA with SuperScript II and oligo(dT)_12–18_ primers (Invitrogen, Thermo Scientific, Illkirch, France) as described previously (12). Quantitative real-time PCR was performed on an ABI Prism 7900HT sequence detection system (Applied Biosystems). TaqMan gene expression assays with TaqMan universal master mix II (Applied Biosystems, Thermo Scientific, Illkirch, France) were used to quantify the expression of *gata3*, *tbet*, *foxp3*, *rorγt*, *ifnγ*, *tnfα*, *il4*, *il10*, and *tgfβ*. Assays were performed in duplicate, and gene expression levels were calculated using the 2^−ΔΔ^*^CT^* method (41), where *C_T_* is the threshold cycle with the TATA box (TaqMan) assay as the reference gene. Fold increases in expression were normalized to expression levels in the sensitized PBS group.

### Evaluation of the fermentative activity of the microbiota.

The concentrations of short-chain fatty acids (SCFAs), i.e., acetate, propionate, butyrate, valerate, caproate, isobutyrate, isovalerate, and isocaproate, were measured in the cecal contents (CC) collected on the day of mouse sacrifice, which were immediately stored at –80°C. Analyses were performed according to the method of Lan et al. (42). After thawing at room temperature, samples were water-extracted, and proteins were precipitated with phosphotungstic acid. The samples were analyzed by gas chromatography using a system (Autosystem; Perkin-Elmer, St. Quentin en Yvelines, France) equipped with a split/splitless injector, a flame ionization detector, and a capillary column (length, 15 m; inside diameter, 0.53 mm; film thickness, 0.5 μm) impregnated with SP100 (Nukol; Supelco, Saint-Quentin-Fallavier, France). The temperatures of the injector, column, and detector were 200°C, 100°C, and 240°C, respectively. The internal standard was 2-ethylbutyrate. All samples were analyzed in duplicate. Data were collected, and peaks were integrated, using Turbochrom software (Perkin-Elmer, Courtaboeuf, France). Results were expressed in micromoles per gram of CC.

### Metabolomic analyses.

Metabolomic analyses were performed by Metabolon (Morrisville, NC, USA) on plasma from sensitized mice (5 to 6 samples per group) according to the procedure described previously (43). Briefly, following methanol extraction, the sample was divided into four fractions: two for analysis by two separate reverse-phase ultraperformance liquid chromatography–tandem mass spectrometry (RP-UPLC–MS/MS) methods with positive-ion-mode electrospray ionization (ESI), one for analysis by RP-UPLC–MS/MS with negative-ion-mode ESI, and one for analysis by hydrophilic interaction liquid chromatography (HILIC)/UPLC–MS/MS with negative-ion-mode ESI. Raw data were extracted, peak-identified, and quality control (QC)-processed using Metabolon’s hardware and software. Compounds were identified by comparison to library entries or to chromatographic and spectral data of purified standards or recurrent unknown entities (>3,300 compounds), using several criteria, such as the retention index and mass-to-charge ratio (*m/z*). With regard to MS/MS spectral data, MS/MS forward and reverse scores were compared between the experimental data and authentic standards. A variety of curation procedures were carried out to ensure the availability of a high-quality data set for statistical analysis and data interpretation. Since each biochemical has its own scale of values, the median concentration of each compound was calculated, and the individual concentrations of the compound determined in the different samples were then divided by this median concentration. In this way, the median value of each compound was set at 1, allowing comparison of the relative concentrations of all the biochemicals on a common scale. Statistical analyses were performed after log transformation and imputation of missing values, if any, using the minimum observed value for each compound.

### Microbiota analyses.

Total bacterial DNA was extracted from the fecal contents of sensitized mice (7 in each probiotic group and 6 in the PBS group) using the DNeasy PowerSoil kit (Qiagen, France). The V3-V4 region of the 16S rRNA gene was amplified for 30 cycles with an annealing temperature of 65°C using *Taq* polymerase MTP (Sigma-Aldrich) and primers PCR1F_460 (CTTTCCCTACACGACGCTCTTCCGATCTACGGRAGGCAGCAG) and PCR1R_460 (GGAGTTCAGACGTGTGCTCTTCCGATCTTACCAGGGTATCTAATCCT). The amplicon lengths were approximately 450 bp (depending on the species). Amplicons were then sent to the Genotoul platform of INRA Toulouse (France) for sequencing. The raw 16S rRNA sequences were analyzed using the bioinformatics pipeline FROGS (Find Rapidly OTU with Galaxy Solution) (44). After quality control depletions, 622,728 total sequences (out of the initial 1,122,764 sequences), comprising 13,835 to 22,836 sequences per sample, were used for analysis. Affiliations were investigated using BLAST (Basic Local Alignment Search Tool) by reference to the SILVA 132 16S database. Data were filtered by retaining only sequences that were present in at least three samples and contributed 0.005% to the microbial community. Only sequences of sufficient quality (alignment of 400 bp and ≥0.95 coverage) were retained. Multiaffiliation was manually checked: each sequence was analyzed with leBiBi (Quick BioInformatic Phylogeny of Prokaryotes), and a single affiliation was accepted based on the results and the phylogenetic tree. The resulting OTU (operational taxonomic unit) table was used for subsequent statistical analysis using R software. The phylogenetic tree was constructed using Mafft and Fasttree on the FROGS pipeline. Samples were standardized to the same depth (13,835 sequences) before analysis.

### Coincubation of the three probiotic strains with mouse bone marrow-derived dendritic cells.

**(i) BMDC generation and probiotic strain preparation.** Dendritic cells were generated from bone marrow collected from the femur and tibia bones of 6- to 8-week-old female BALB/cByJ mice as described previously by Lutz et al. (45). Briefly, after red cell lysis, bone marrow cells were seeded in bacteriological petri dishes containing 10 ml of Iscove’s modified Dulbecco’s medium (IMDM) with added streptomycin and penicillin (all from Gibco Thermo Fisher, France), 10% heat-inactivated fetal calf serum (PAN Biotech, Dutscher, France), β-mercaptoethanol (50 μM; Sigma-Aldrich, France), and 20 ng/ml murine recombinant GM-CSF (Peprotech, Neuilly-sur-Seine, France). Freshly prepared medium was added every 3 days, and BMDCs were used on D9 of culture. The phenotype of BMDCs was characterized according to the expression of cell surface markers, in particular CD11c, determined by FACS (fluorescence-activated cell sorting) flow cytometry analysis.

Bacterial strains were prepared as described previously (12). Briefly, they were grown in Man, Rogosa and Sharpe (MRS) medium with cysteine under anaerobic conditions (CO_2_-H_2_-N_2_, 10:10:80) at 37°C up to 1 h after the start of the stationary phase. They were then washed and frozen in PBS containing 15% glycerol at –80°C until the coincubation experiments. The number of live bacteria (expressed as CFU) was estimated by culture using a Whitley automated spiral plater (WASP; AES-Chemunex, Bruz, France). Three independent growth experiments were performed for each strain.

**(ii) Bacterial cell coincubation.** At D9, BMDCs were seeded at 3 × 10^5^/well and incubated for 18 to 20 h with medium alone, TLR-2 and TLR-9 agonists (PAM2CSK4 and CpG-ODN 1825, each at 0.5 μg/well), or a single probiotic strain (multiplicity of infection [MOI], 50:1). Cells were then collected by gentle pipetting and were centrifuged for 10 min at 300 × *g* for FACS analysis. Culture supernatants were collected and stored at –20°C for IL-10 measurement (ELISA, BD OptEIA; BD Biosciences, France).

For FACS analysis of surface marker expression, cells were resuspended in cold PBS containing 1% (vol/vol) fetal calf serum. For all staining experiments, cells were incubated with Fc receptor-blocking anti-CD32 (2.4G2). Phycoerythrin (PE)-conjugated anti-CD11c, fluorescein isothiocyanate (FITC)-conjugated MHC-II, PE-Cy7-conjugated anti-CD80, and allophycocyanin (APC)-conjugated CD86 monoclonal antibodies (MAb) and appropriate isotypic control MAb were purchased from eBiosciences, Paris, France. Cells were incubated with the appropriate MAb for 30 min on ice, then washed with 3 ml PBS-fetal bovine serum (FBS), and finally resuspended in 300 μl of 1% paraformaldehyde for flow cytometric analysis using a FACS-Accuri C6 flow cytometer (BD Biosciences). 7-Aminoactinomycin D (7-AAD) was used for staining to differentiate live and dead cells.

### Statistical analysis.

Results for allergic and sensitization markers, cytokines, and metabolites are expressed as medians [ranges]. Gene expression, surface markers of BMDC, and IL-10 production by BMDCs are expressed as means ± standard errors of the means (SEM). Results were analyzed using the nonparametric Kruskal-Wallis and Mann-Whitney U tests. Differences were considered to be statistically significant when the *P* value was <0.05. Data were analyzed using SPSS 20 software (Statistical Package for the Social Sciences; IBM, France).

For microbiota analyses, the normalized OTU table was analyzed using the Phyloseq R package to study intra- and intersample diversity (α- and β-diversity) (46). α-Diversity was studied by calculating the number of observed OTUs and the Shannon index. Statistical differences between groups were determined using Kruskal-Wallis and Mann-Whitney U tests. β-Diversity was studied using several distances (Jaccard, Bray-Curtis, and UniFrac) to analyze compositional diversity. Differences between groups were evaluated by multivariate analysis of permutation variances using distance matrices (PERMANOVA) and constrained analysis of principal coordinates (CAP). The LefSe pipeline (linear discriminant analysis [LDA] effect size) (47) was used to identify putative probiotic-related microbiota biomarkers. The LefSe algorithm uses a nonparametric factorial Kruskal-Wallis rank sum test, an unpaired Wilcoxon rank sum test, and LDA to estimate the effect size of each differentially abundant OTU; the threshold of statistical significance (α parameter) was set at 0.05 for the Kruskal-Wallis and Wilcoxon tests and at 2.0 for the logarithmic score of LDA analysis. SIMPER (similarity percentage procedure) analyses (48) were performed at the genus level. Ecologically organized heat maps were constructed using CAP ordination and Bray-Curtis dissimilarity (49).

## Supplementary Material

Supplemental file 1
