# Supplementary material for: Three Candidate Probiotic Strains Impact Gut Microbiota and Induce Anergy in Mice with Cow's Milk Allergy
Source: Appl Environ Microbiol. 2020 Oct 15;86(21):e01203-20. doi: 10.1128/AEM.01203-20 (PMC7580549; doi:10.1128/AEM.01203-20)
Supplement: Supplemental file 1 [file AEM.01203-20-s0001.pdf]

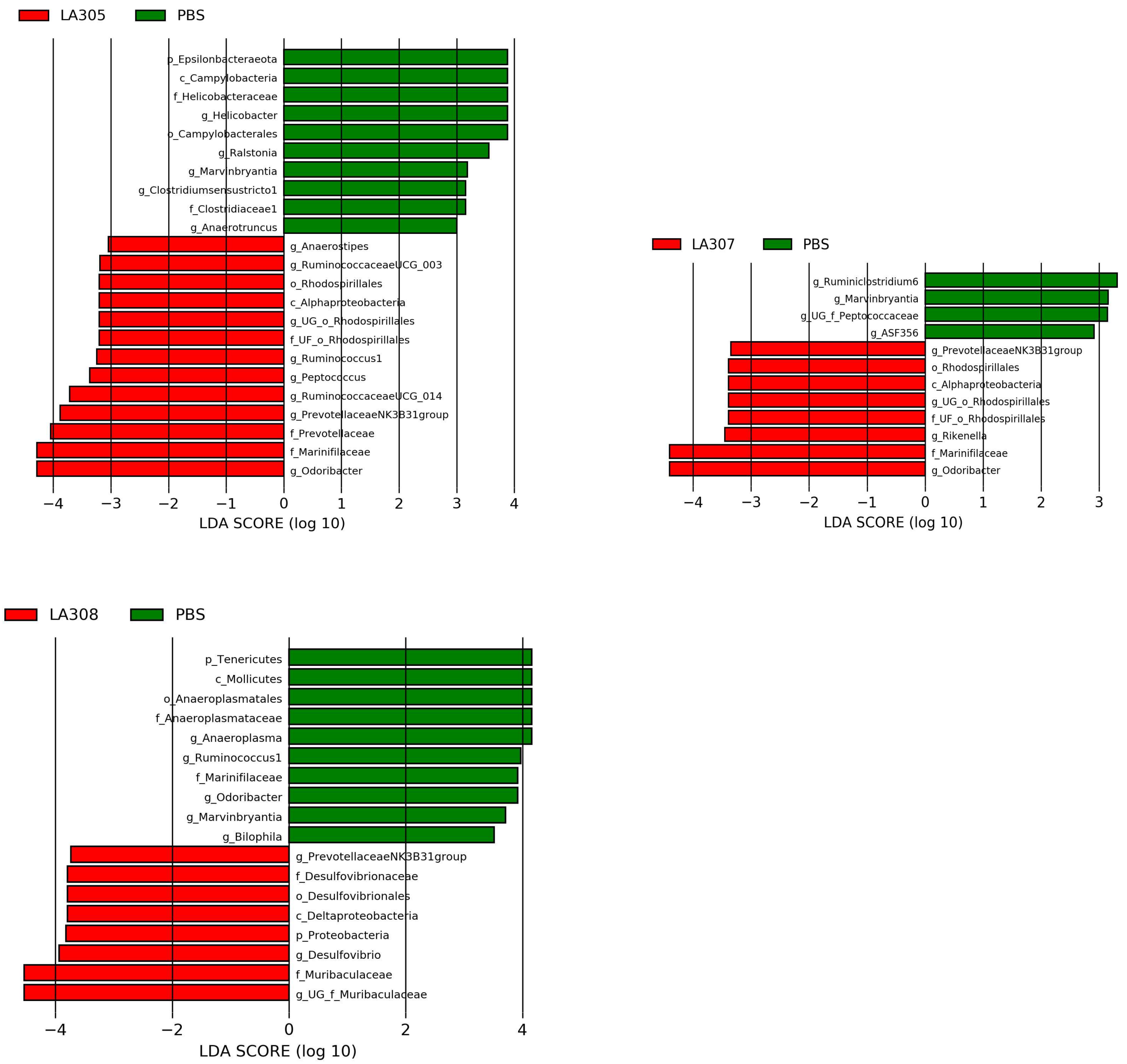

FIG. S1: Lefse analyses of cecal microbiota

Graphs of linear discriminant analysis (LDA) effect size (LEfSe). Comparison of each probiotic group to PBS-treated mice (n=6-7/ group). Horizontal bars represent the effect size for each taxon. The length of the bar represents the log10 transformed LDA score, indicated by vertical dotted lines. Microbiota organisms affected by probiotics are indicated in red, those affected by PBS in green. The threshold on the logarithmic LDA score for discriminative features was set to 2.0. The taxon of bacteria with statistically significant change ($p < 0.05$) in the relative abundance is written alongside the horizontal lines. The name of the taxon level is abbreviated as p—phylum; c—class; o—order; f—family, and g—genus.

Table S1: Differences in fecal microbiota between probiotic treated-mice and PBS-treated ones using Simper analysis. Average of abundance for Probiotic (Av. Probiotic) and PBS (Av. PBS) groups are indicated.

Strain	Family	Genus	Av.Probiotic	Av.PBS	p
LA305	*Marinifilaceae*	*Odoribacter*	1104.00000	429.6667	0.01039896
LA305	*Prevotellaceae*	*Prevotellaceae NK3B31 group*	278.00000	1.8333	0.00149985
LA305	*Helicobacteraceae*	*Helicobacter*	0.00000	266.0000	0.00069993
LA305	*Ruminococcaceae*	*Ruminococcaceae UCG-014*	241.85714	45.0000	0.00909909
LA305	*Ruminococcaceae*	*Ruminococcus 1*	62.85714	7.1667	0.01479852
LA305	*Lachnospiraceae*	*Marvinbryantia*	11.28571	51.5000	0.00429957
LA305	*Deferribacteraceae*	*Mucispirillum*	6.14286	39.1667	0.02269773
LA305	*Clostridiaceae 1*	*Clostridium sensu stricto 1*	0.28571	9.8333	0.01669833
LA305	*Ruminococcaceae*	*Ruminococcaceae UCG-003*	8.42857	2.3333	0.03059694
LA305	*Peptococcaceae*	*unknown genus*	1.85714	4.8333	0.03529647
LA305	*Peptococcaceae*	*Peptococcus*	6.28571	3.1667	0.01979802
LA305	*Burkholderiaceae*	*Ralstonia*	1.85714	3.8333	0.04939506
LA307	*Marinifilaceae*	*Odoribacter*	1308.143	429.6667	0.0122988
LA307	*Prevotellaceae*	*Prevotellaceae NK3B31 group*	65.857	1.8333	0.0078992
LA307	*Lachnospiraceae*	*Marvinbryantia*	15.714	51.5000	0.0175982
LA307	*Peptococcaceae*	*unknown genus*	2.000	4.8333	0.0312969
LA308	*Lachnospiraceae*	*Lachnospiraceae NK4A136 group*	4152.7143	2521.5000	0.04729527
LA308	*Anaeroplasmataceae*	*Anaeroplasma*	48.4286	474.0000	0.00389961
LA308	*Desulfovibrionaceae*	*Desulfovibrio*	306.1429	18.5000	0.03709629
LA308	*Marinifilaceae*	*Odoribacter*	209.7143	429.6667	0.00149985
LA308	*Prevotellaceae*	*Prevotellaceae NK3B31 group*	105.7143	1.8333	0.01159884
LA308	*Desulfovibrionaceae*	*Bilophila*	15.1429	75.0000	0.00859914
LA308	*Lachnospiraceae*	*Marvinbryantia*	8.2857	51.5000	0.00039996
LA308	*Ruminococcaceae*	*Ruminococcus 1*	1.2857	7.1667	0.04539546

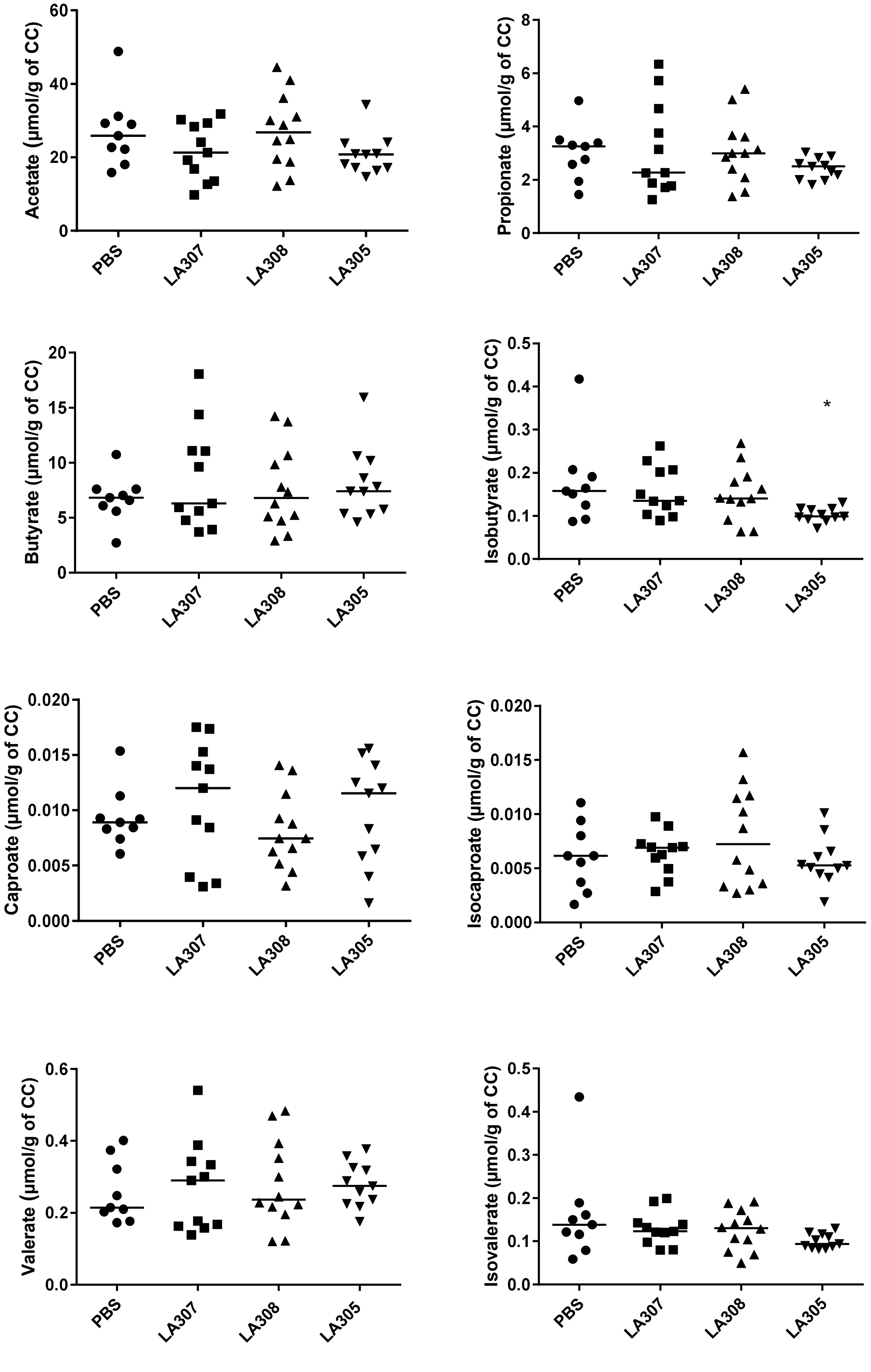

FIG. S2: Impact of probiotic strains on short-chain fatty acids (SCFAs).
Cecal contents (CC) were collected on Day 63 from sacrificed WP-sensitized mice (PBS group: n=9; probiotic-treated groups; n=12 per probiotic) and SCFAs were measured by gas-liquid chromatography. Each point represents a mouse, the central horizontal line representing the median value for each group. Data were compared to WP-sensitized mice treated with PBS using the Mann-Whitney U test, *P<0.05
